# A novel method of tunneling retroperitoneoscopic adrenalectomy: a prospective study

**DOI:** 10.1186/s12894-024-01484-x

**Published:** 2024-04-30

**Authors:** Pengcheng Zhang, Yuhan Pei, Yunlai Zhi, Fanghu Sun

**Affiliations:** Department of Urology, Lianyungang Clinical College of Nanjing Medical University, The First People’s Hospital of Lianyungang, 6 Zhenhua East Road, Lianyungang, 222000 China

**Keywords:** Adrenalectomy, Retroperitoneoscopic surgery, Tunneling

## Abstract

**Background:**

To introduce the surgical technique and our team’s extensive experience with tunnel method in laparoscopic adrenalectomy.

**Methods:**

From July 2019 to June 2022, we independently designed and conducted 83 cases of " Tunnel Method Laparoscopic Adrenalectomy,” a prospective study. There were 45 male and 38 female patients, ages ranged from 25 to 73 years(mean: 44.6 years).The cases included 59 adrenal cortical adenomas, 9 pheochromocytomas, 6 cysts, 4 myelolipomas, 1 ganglioneuroma, and 4 cases of adrenal cortical hyperplasia. In terms of anatomical location, there were 39 cases on the left side, 42 on the right side, and 2 bilateral cases. Tumor diameters ranged from 0.6 to 5.9 cm(mean: 2.9 cm). Utilizing ultrasound monitoring, percutaneous puncture was made either directly to the target organ or its vicinity, and the puncture path was manually marked. Then, under the direct view of a single-port single-channel laparoscope, the path to the target organ in the retroperitoneum or its vicinity was further delineated and separated. This approach allowed for the insertion of the laparoscope and surgical instruments through the affected adrenal gland, thereby separating the surface of the target organ to create sufficient operational space for the adrenalectomy.

**Results:**

All 83 surgeries were successfully completed. A breakdown of the surgical approach reveals that 51 surgeries were done using one puncture hole, 25 with two puncture holes, and 7 with three puncture holes. The operation time ranged from 31 to 105 min (mean: 47 min), with a blood loss of 10 to 220mL (mean: 40 mL). Notably, there were no conversions to open surgery and no intraoperative complications. Postoperative follow-up ranged from 6 to 28 months, during which after re-examination using ultrasound, CT, and other imaging methods, there were no recurrences or other complications detected.

**Conclusions:**

The completion of the tunnel method laparoscopic adrenalectomy represents a breakthrough, transitioning from the traditional step-by-step separation of retroperitoneal tissues to reach the target organ in conventional retroperitoneoscopic surgery. This method directly accesses the target organ, substantially reducing the damage and complications associated with tissue separation in retroperitoneoscopic surgery, As a result, it provides a new option for minimally invasive surgery of retroperitoneal organs and introduces innovative concepts to retroperitoneoscopic surgery.

## Background

Laparoscopic adrenalectomy is the standard procedure for treating adrenal tumors. With the widespread implementation of retroperitoneoscopic adrenalectomy, there has been a notable accumulation of surgical experience. Alongside, a deeper understanding of the anatomy of the retroperitoneal space has also facilitated the procedure’s further refinement. As a result, the steps of retroperitoneoscopic adrenalectomy have been standardized [1, 2], initiating with the establishment of an adequate retroperitoneal space (via balloon or instrument separation), followed by the layer-by-layer separation and expansion of the retroperitoneal tissues based on inherent landmarks. This process involves clearing some fatty tissue and mobilizing the upper pole of the kidney to expose the target organ (adrenal gland or its tumor), thereby facilitating laparoscopic observation and instrument manipulation; ultimately, the surgery is completed by separating along the target organ. It’s important to note that during this process, the separation of normal tissues in the retroperitoneum inevitably causes damage and may lead to complications; specifically, the larger the area of separation, the greater the damage. To minimize separation damage during the exposure of the adrenal gland, we innovated with a “targeted retroperitoneoscopic adrenalectomy” that precisely advances to the target organ in the retroperitoneum, namely the adrenal gland or its tumor, under the direct vision of a single-port, single-channel retroperitoneoscope, guided by manual marked navigation. However, this approach has limitations due to the operative equipment and the relative difficulty of the procedure, making it more suitable for smaller adrenal adenomas. For larger tumors and pheochromocytomas, which are prone to bleeding, further development is needed, as it does not offer advantages in operation time. Building on this insight, we designed the tunnel method for the retroperitoneoscopic technique and applied it to retroperitoneoscopic adrenalectomy. From July 2019 to June 2022, 83 cases were completed with satisfactory results, as reported below.

## Methods

### Clinical information

This group included 83 cases: 45 male and 38 female. Ages ranged from years(mean: 44.6 years). There were 39 cases on the left side, 42 on the right side, and 2 bilateral cases. Tumor diameters ranged from 0.6 to 5.9 cm (mean: 2.9 cm). Preoperatively, 59 cases were diagnosed with adrenal cortical adenoma (41 cases of primary aldosteronism, 7 with Cushing’s syndrome, 11 non-functional adenomas), 9 with pheochromocytoma, 6 cysts, 4 myelolipomas, 1 ganglioneuroma, and 4 cases of adrenal cortical hyperplasia. All patients underwent ultrasound, CT, or MRI imaging for preoperative localization and diagnosis, ensuring precise targeting and planning for the surgical procedure. For those exhibiting preoperative blood pressure and heart rate abnormalities, preparations were meticulously undertaken to achieve stabilization before scheduling surgery, thereby mitigating potential perioperative risks.

Inclusion criteria for the cases were: (1) benign adrenal lesion; (2) body mass index (BMI) < 30; (3) maximum tumor diameter < 6 cm; (4) no severe cardiopulmonary comorbidities; ⑤ classified as ASA (American Society of Anesthesiologists) Grade I or II.

**Fig. 1:**
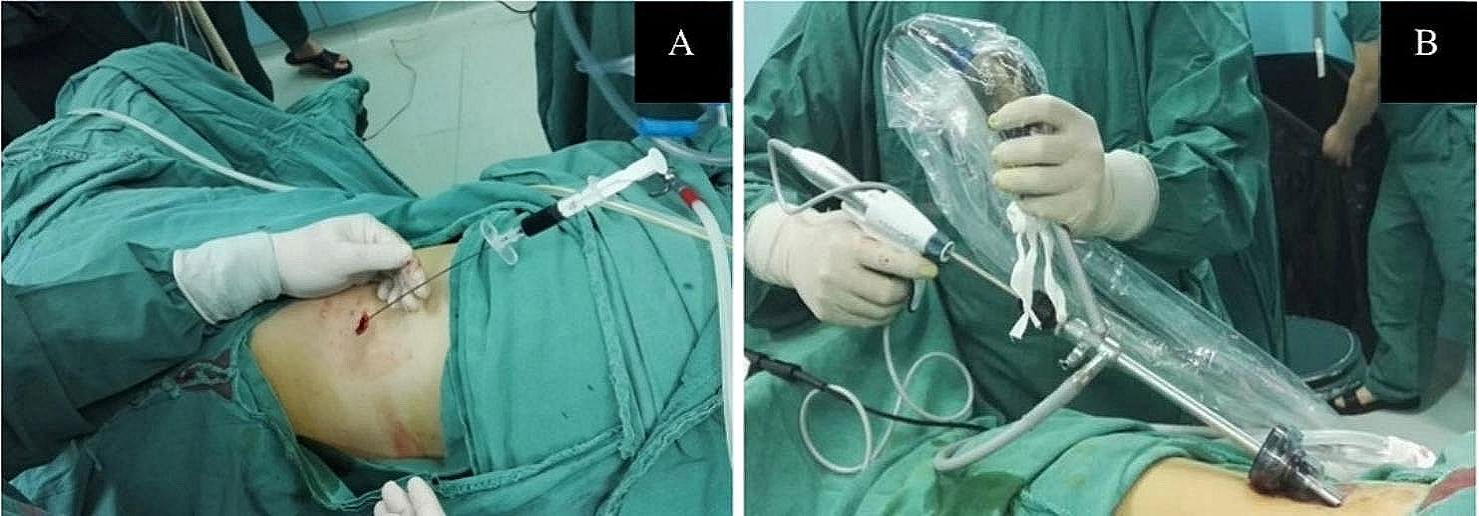
Injection of methylene blue solution for localization (**A**); Insertion of a 0° lateral-view laparoscope (**B**)

Laparoscopic Instruments: American Ethicon GEN11 5 mm*360 mm ultrasonic knife, along with the matching 5 mm*330 mm curved dissecting forceps. Side-view 0° laparoscope (with working channel, diameter 6 mm) model: Karl Storz 26,034 V 0° laparoscope (manufacturer: Karl Storz, Germany).Part Number: 26034v, Manufacturer: Karl Storz, Diameter: 10.0 mm, Angle: 0º, Working Length: 27 cm (Fig. 1A, B).

### Operation procedure

**Fig. 2:**
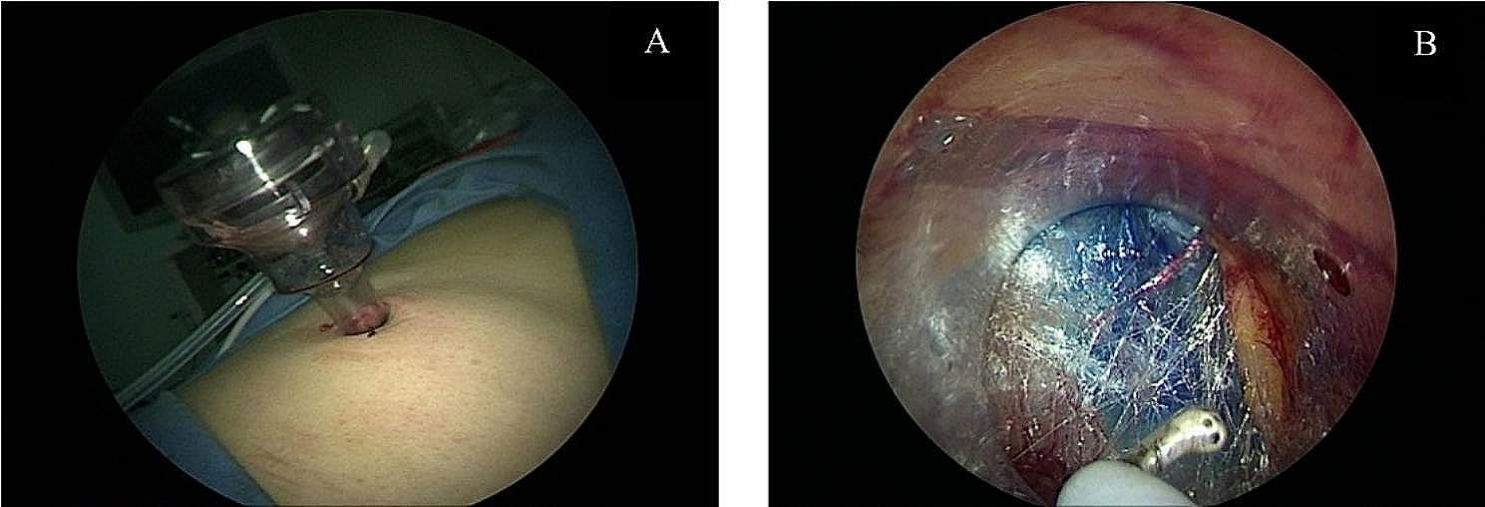
Insertion of the sheath by the closed method (**A**); Fatty tissue connected by semi-transparent connective tissue. Under direct vision, the methylene blue marking was located (**B**)

**Fig. 3:**
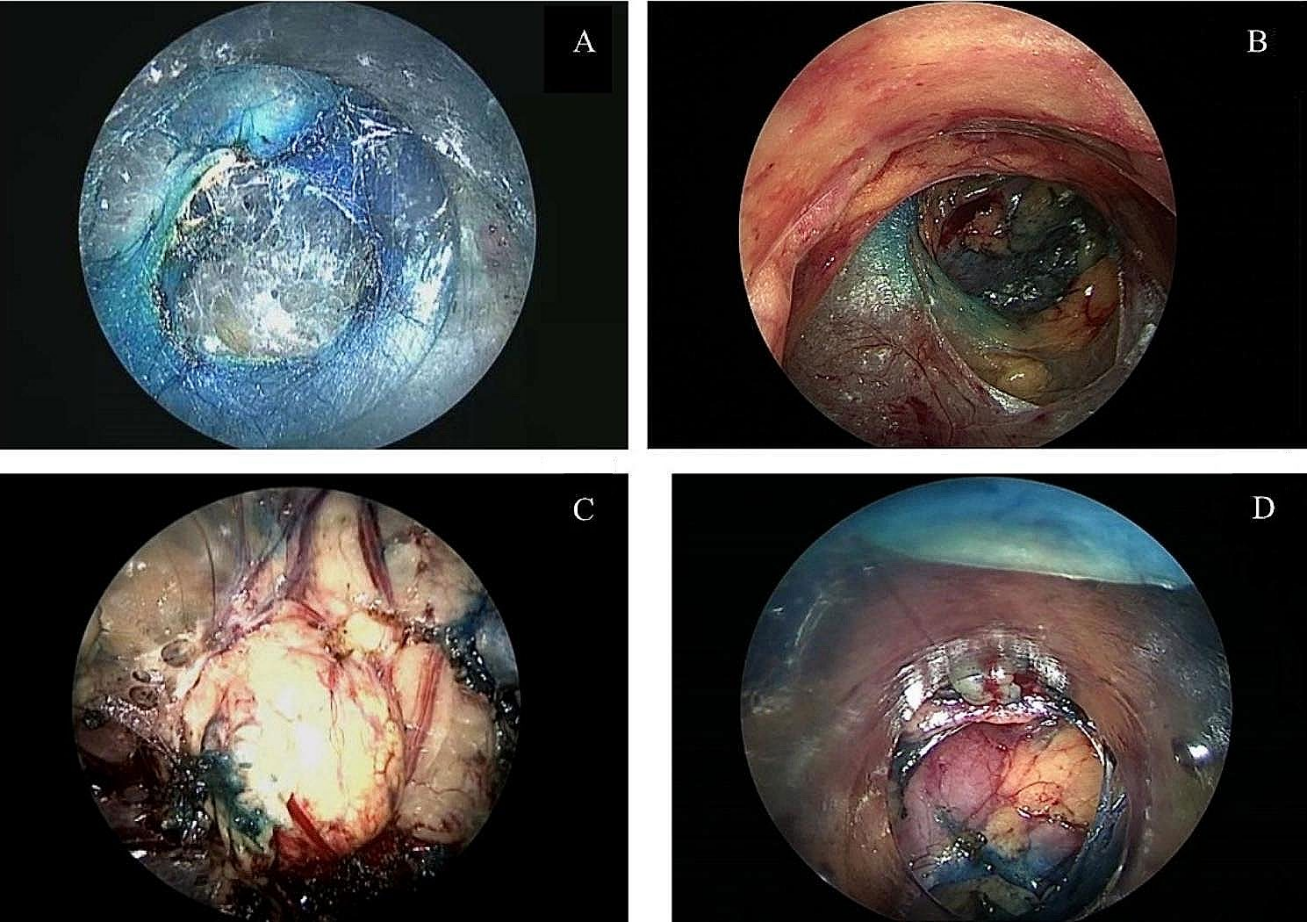
Dissect along the methylene blue markings to the Gerota’s fascia and create a small opening (**A**). Continue the dissection to form a tunnel (**B**) leading to the adrenal tumor (**C**) or to the surface of the upper pole of the kidney (**D**)

**Fig. 4:**
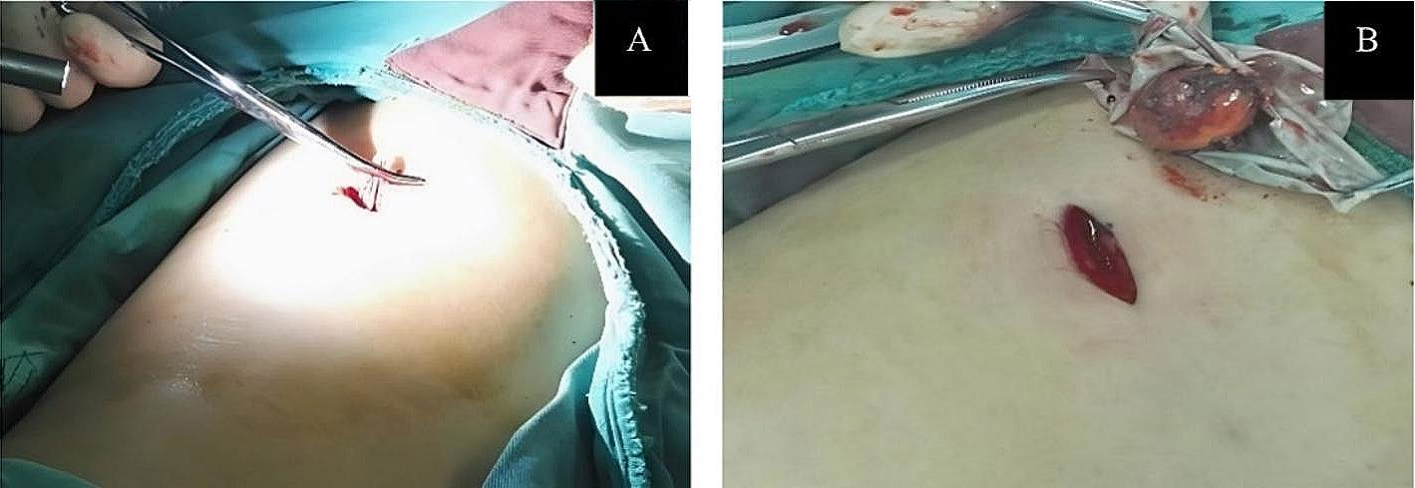
Pull out one end of the specimen bag through the incision (**A**), and extract the specimen (**B**)

**Fig. 5:**
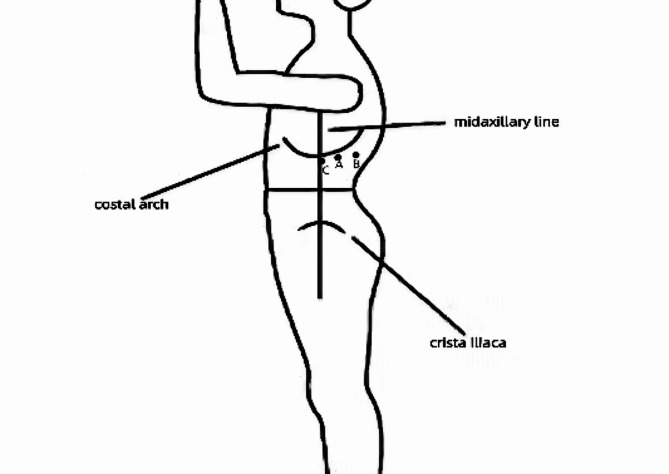
Make a transverse incision of 1.2 cm at the posterior axillary line beneath the twelfth rib (Point **A**). The puncture hole is located approximately 5 cm medial to Point A, 1–3 cm beneath the twelfth rib (Point **B**), along the midaxillary line below the rib margin (Point **C**)

General anesthesia was administered via endotracheal intubation, and the patient was carefully positioned in a lateral position with a slightly elevated waist bridge to optimize surgical access. After disinfection and draping, a 1.2 cm transverse skin incision was made below the twelfth rib along the posterior axillary line (Point A) (Fig. 2). Ultrasound scanning was performed at this point, and upon locating the adrenal tumor, an 18G puncture needle (later changed to a Veress needle) was inserted towards the adrenal tumor to reach its capsule. If it was difficult to locate the adrenal tumor with ultrasound, the puncture needle could be inserted to the posterior lateral surface of the upper pole of the kidney under ultrasound guidance. While injecting methylene blue solution, the needle was withdrawn (injecting 0.5 ml of methylene blue solution diluted to 0.5 mg/ml for every 0.5 cm of withdrawal). Using a single-port, single-channel laparoscope under direct vision, manually marked separations were made to form a channel to the target organ in the retroperitoneum or its vicinity. Closed puncture technique [3] was used to enter the retroperitoneal space(a 10 mm disposable sheath puncture instrument manufactured by Hangzhou Kangji Medical Instruments) (Fig. 3A).The pneumoperitoneum pressure was maintained at 10 ~ 15 mmHg (1 mmHg = 0.133 kPa). Under pneumoperitoneum pressure, the retroperitoneal space revealed fatty tissue connected by semi-transparent connective tissue (Fig. 3B). Under direct vision, the methylene blue marking was located (Fig. 3B), and the sheath was advanced deeper along it: cutting through a small amount of semi-transparent connective tissue, creating a space sufficient for the passage of the sheath. Upon reaching the Gerota’s fascia, a small hole was opened (Fig. 4A), and the laparoscope was passed through, continuing to advance forward and separate to form a retroperitoneal tunnel (Fig. 4B), leading directly to the adrenal tumor (Fig. 4C). If the laparoscope was advanced along the marking to the surface of the upper pole of the kidney (Fig. 4D), the adrenal gland and tumor were located by separating the fatty tissue above the upper pole of the kidney. The tumor was separated along its surface and removed. Alternatively, the inferior part of the adrenal gland was separated to locate the central adrenal vein, which was clamped, cut, and then continued to be separated along the adrenal capsule until the adrenal gland was completely freed. If the operation was found to be difficult, additional puncture holes could be made for assistance. The puncture hole was located about 5 cm posterior and medial to Point A, 1–3 cm below the twelfth rib (Point B) (Fig. 2), into which a 5 mm Trocar was inserted. If difficulties persist, retract the laparoscope to below the abdominal wall, separate towards the inside along the inner side of the abdominal wall, and insert a 5–10 mm Trocar below the mid-axillary line at the rib margin (Point C) (Fig. 2), appropriately enlarging the tunnel to facilitate operations at Point C. Place the specimen in a homemade specimen bag for removal. Method for Creating the Specimen Bag: First, take a sterile rubber medical glove and tightly roll it at the base of the thumb’s distal joint. Next, ligate it with two turns of size 7 silk suture, ensuring a spacing of 3 mm between the two turns. Then, cut off the five finger sections of the glove below the ligature to create a specimen bag. This results in a bag that is 16 cm long with an inner diameter of 6 cm. The surgeon uses dissecting forceps to grasp the bottom of the glove, places it into the retroperitoneum, places the cut specimen into the specimen bag, clamps the forceps to prevent detachment, removes the 10 mm trocar, clamps the glove with vascular forceps to close it (Fig. 5a), and retrieves the specimen (Fig. 5b).After placing a drainage tube in the incision, withdraw all the trocars. Suture the incision.

## Results

All 83 surgeries were successfully completed. A breakdown of the approach used reveals that 51 surgeries utilized one puncture hole, 25 employed two puncture holes, and 7 required three puncture holes. The operation time ranged from 31 to 105 min (mean: 47 min), with a blood loss of 10 to 220mL (mean: 40 mL). There were no conversions to open surgery and no intraoperative complications. The postoperative peritoneal drainage tubes were removed after 1 to 4 days, and the average postoperative hospital stay was 2 to 6 days(mean: 3.5 days) (Table 1). In terms of follow-up, the period ranged from 6 to 28 months, during which, after re-examination using ultrasound, CT, and other imaging methods, no recurrences or other complications were detected.

**Table 1:** Preoperative and postoperative status of the patient

Parameter	Data
Age (years)	44.60 ± 6.94
Male/Female (n)	45/38
Body Mass Index (kg/m²)	26.41 ± 4.56
Surgical duration/min	47.00 ± 6.79
Hospital Stay/days	3.50 ± 1.34
Time to Eating /h	12.02 ± 4.27
Time to First Ambulation /h	18.97 ± 5.71
Blood Loss/mL	40.00 ± 5.83
Postoperative VAS Score/points	3.56 ± 0.52
Subcutaneous Emphysema	0
Incision Length/cm	3.18 ± 0.36
Puncture Holes	
1	51
2	25
3	7

## Discussion

Adrenal diseases, such as adrenal tumors and adrenal hyperplasia, are common and frequently occurring conditions in urological surgery. With the development and widespread use of diagnostic technologies like CT scans, an increasing number of patients are being diagnosed [4]. Most patients with adrenal tumors and some with adrenal hyperplasia require adrenalectomy to alleviate their conditions. Therefore, adrenalectomy has become one of the most common surgeries in urology. The pursuit of minimizing surgical trauma and complications marks a significant trend in the evolution of surgical practices. In the late 20th century, the introduction of laparoscopy revolutionized surgical procedures, ushering in a new era beyond traditional open surgery. Minimally invasive surgery, particularly laparoscopic surgery, has established its superiority, demonstrating promising prospects for the future. With advancements, urological laparoscopic techniques have gained widespread acceptance and application. Retroperitoneoscopic techniques, benefiting from their retroperitoneal approach, offer direct access to the unilateral adrenal gland and urinary system, thus gaining increasing attention for causing less disturbance to intra-abdominal organs, ensuring shorter operation times, and facilitating faster recovery. However, retroperitoneal surgeries present challenges such as relatively smaller operating spaces, less clear anatomical landmarks, and greater operational difficulty, compared to transabdominal laparoscopic surgeries. With the continuous improvement of operators’ technical experience, advancements in surgical instruments, and the adoption of new techniques, retroperitoneoscopic adrenalectomy has demonstrated significant advantages, establishing it as the gold standard for adrenal gland surgery treatment​​ [5, 6], making it one of the most performed urological laparoscopic surgeries both domestically and internationally.

With the widespread implementation of retroperitoneoscopic adrenalectomy, there has been a notable increase in surgical experience. Additionally, a deeper understanding of the anatomy of the retroperitoneal space has also facilitated further refinement of the procedure. Currently, the procedural steps for retroperitoneoscopic adrenalectomy have been largely standardized [2, 8]​​: It begins with creating sufficient retroperitoneal space (using balloon dissection or instrumentation), followed by separating and expanding the retroperitoneal tissues layer by layer, based on internal landmarks. This includes clearing part of the fatty tissue and freeing the upper pole of the kidney, which aids in revealing the target organ (adrenal gland or tumor) for easier laparoscopic visualization and instrument handling. The final step is dissecting along the target organ to complete the surgery. Analyzing the above surgical process from an anatomical perspective, apart from the smaller abdominal incision, the operations within the retroperitoneum are similar to those of open surgery, or it can be said to replicate the procedural steps of open surgery​​ [4–8]. Thus, the trauma caused by retroperitoneoscopic adrenalectomy, compared to traditional open surgery, is similar in terms of internal damage in the retroperitoneum, aside from the smaller abdominal wall incision. However, this approach can lead to corresponding complications and accidental injuries, and the lack of direct tactile feedback for the surgeon may increase the risk of accidental injuries. In recent years, to further reduce surgical trauma and improve cosmetic outcomes, single-port laparoscopic techniques have rapidly developed and emerged as a significant area of clinical research [2]​​. This has even led to the emergence of robotic assistance for single-port laparoscopic surgeries. Single-port retroperitoneoscopic adrenalectomy is also being performed internationally, reducing the abdominal wall incisions from the original 3–4 ports to a single larger port, which has a better cosmetic effect. This type of surgery creates a similar space for separation of tissues in the retroperitoneum as traditional retroperitoneoscopic surgery​​ [2], thus causing similar trauma and complications. Moreover, it requires a larger single port, the total length of which is comparable to the incision in traditional retroperitoneoscopic surgery. To overcome the trauma caused by this surgical approach, it is essential to explore more minimally invasive surgical methods, which will undoubtedly become a new research direction in laparoscopic surgery. According to reports [7, 9, 10]​​, the complication rate of retroperitoneoscopic adrenal surgery is about 10%, with more severe complications occurring at a rate of 2–5.7%. Common complications include peritoneal injury, pleural injury, injury to the central adrenal vein, severe hypercapnia, wound infection, and retroperitoneal hematoma. Additionally, there are several reports of serious complications such as injury to the inferior vena cava, renal vessels, liver, spleen, pancreas, and massive bleeding leading to patient death. All the above complications can occur during the process of separating and locating the adrenal gland or adrenal tumor (the target organ)​​ [2, 8, 9]. Therefore, reducing the complications and accidental injuries in retroperitoneoscopic adrenal surgeries requires more precise operations and technical support, which will continue to be a major focus in clinical research for this surgery.

Reducing trauma and complications in laparoscopic surgery has become a research focus in recent years. This interest has been sparked by the need to improve patient outcomes and minimize post-operative recovery times. Recently, the use of surgical navigation systems and 3D printing technology has emerged as innovative methods to guide surgical procedures, showing good results in avoiding vital organs and reducing accidental injuries [11–13]. However, these advances have not improved the steps involved in operating within the retroperitoneum or the extent of tissue separation. Our recently developed “targeted retroperitoneoscopic adrenalectomy” has, through initial clinical application, proven capable of more directly accessing the adrenal gland. With a personalized design of the retroperitoneal approach, it can reduce trauma to the retroperitoneum, enhance surgical safety, and achieve better cosmetic results. However, due to the limitations of the operative equipment and the relative difficulty of the procedure, this technique is more suitable for smaller adrenal adenomas. For larger tumors and pheochromocytomas, which are prone to bleeding, further development is needed, as currently, there is no advantage in terms of operation time.

Our developed “targeted retroperitoneoscopic adrenalectomy” utilizes manual marked navigation to precisely guide a single-port, single-channel retroperitoneoscope directly to the target organ within the retroperitoneum, namely the adrenal gland or its tumor. The successful implementation of the tunnel method retroperitoneoscopic adrenalectomy, after preliminary clinical application, has shown that this technique achieves the experimental design objectives, namely, more direct access to the adrenal gland and other target tissues, effectively reducing separation trauma and related complications.

Intraoperative Precautions:1. Due to the limited operative space, handling complex cases such as larger adrenal tumors or obese patients can be challenging, necessitating additional puncture holes. Pheochromocytomas, which are rich in blood vessels and often adhere to surrounding tissues, can cause significant fluctuations in blood pressure during surgery. Extreme caution is required, and additional puncture holes may be needed when beginning to separate the tumor, with preparations for switching to traditional laparoscopy if necessary. Before the surgery, the patient’s blood pressure and heart rate need to be stabilized, and then the surgery can be scheduled. Moreover, the patient’s recovery after surgery is relatively slow, and the longest hospital stay can be up to 6 days.2. As the direction of the trocar’s puncture slightly differs from that of the puncture needle, and anatomical changes are likely to occur after insufflating the retroperitoneum, the methylene blue artificial markings may be lost after the laparoscope is inserted. Generally, a slight separation in the forward and upward direction under the abdominal wall can help locate the artificial markings. 3. Commercially available methylene blue solutions are too concentrated and should be diluted tenfold to prevent the overly deep color from impairing the operative field of view.

## Conclusions

The accomplishment of our self-designed tunnel method retroperitoneoscopic adrenalectomy represents a breakthrough over the conventional retroperitoneoscopic surgery, which requires layer-by-layer separation of retroperitoneal tissues to form a surgical field and then reach the target organ. This method reduces the trauma and complications of retroperitoneoscopic surgery, offering a new option for minimally invasive surgery of retroperitoneal organs and introducing fresh perspectives to retroperitoneoscopic procedures. Due to the limited time and relatively small number of cases, the long-term effects of this technique require further evaluation and research through clinical practice.

## Data Availability

All data generated or analysed during this study are included in this published article.

## Abbreviations

CT: Computed tomography

## References

[1] Chih-Chin Yu,Yao-Chou Tsai (2017). Current surgical technique and outcomes of laparoendoscopic single-site adrenalectomy. Urol Sci.

[2] Wen S-HCC-NHS-C et al. Laparoendoscopic single-site retroperitoneoscopic adrenalectomy compared with conventional laparoscopy and open surgery. Urol Sci 2016 28(1),36–41.

[3] Conzo G, Tartaglia E, Gambardella CD, et al. Minimally invasive approach for adrenal lesions: systematic review of laparoscopic versus retroperitoneoscopic adrenalectomy and assessment of risk factors for complications. Int J Surg. 2016;28:118–23. 10.1016/j.ijsu.2015.12.042 26708860

[4] Carlos E. Costa Almeida,Teresa Caroço,posterior retroperitoneoscopic adrenalectomy—case series. Int J Surg Case Rep 2018,51:174–7. 10.1016/j.ijscr.2018.08.044 PMC6122227 30173077

[5] Nicola PRAH, Lee et al. Impact of novel techniques on minimally invasive adrenal surgery: trends and outcomes from a contemporary international large series in urology. World J Urol 2016,34(10):1473–9. 10.1007/s00345-016-1791-9 26923920

[6] Andreas Kiriakopoulos,Athanassios Petralias,Dimitrios Linos. Posterior retroperitoneoscopic versus laparoscopic adrenalectomy in sporadic and MENIIA pheochromocytomas.Surgical Endoscopy,2015, 29 (8):2164–70. 10.1007/s00464-014-3912-0 25303922

[7] Sun F, FU B, KE M et al. Retroperitoneoscopic adrenalectomy in semilateral supine position[J]. Chin J Urol, 2011: 509–11.

[8] Antonio AG,Fabrizio, Di Maida R, Tellini et al. Robot-assisted partial nephrectomy with 3D preoperative surgical planning: video presentation of the florentine experience. International Braz J Urol. 2021;47 (6):1272–1273. 10.1590/S1677-5538.IBJU.2020.1075 PMC8486435 34156192

[9] Young Jun Chai,Jung-Woo Woo,Hyungju Kwon et al. Comparative outcomes of lateral transperitoneal adrenalectomy versus posterior retroperitoneoscopic adrenalectomy in consecutive patients: A single surgeon’s experience. Asian J Surg 2016,39(2):74–80. 10.1016/j.asjsur.2015.04.005 26117204

[10] Ibrahim Halil Bozkurt,Murat Arslan,Tarik Yonguc et al. Laparoscopic adrenalectomy for large adrenal masses: is it really more complicated? Kaohsiung J Med Sci 2015,31(12):644–8. 10.1016/j.kjms.2015.09.005 PMC11916311 26709227

[11] Kusaka M, Sugimoto M, Fukami N (2015). Initial experience with a tailor-made Simulation and Navigation Program using a 3-D printer model of kidney transplantation surgery. Transpl Proc.

[12] Yu-Mi Ryang,Jimmy Villard,Thomas Obermüller,Benjamin Friedrich,Petra Wolf,Jens Gempt,Florian Ringel,Bernhard Meyer. Learning curve of 3D fluoroscopy image–guided pedicle screw placement in the thoracolumbar spine. Spine J 2015,15(3):467–76. 10.1016/j.spinee.2014.10.003 25315133

[13] Sun F, FU B, KE M (2013). Single-port retroperitoneoscopic decortication for renal cysts [J]. Chin J Urol.

